# Determination of Minerals in Soft and Hard Cheese Varieties by ICP-OES: A Comparison of Digestion Methods

**DOI:** 10.3390/molecules28103988

**Published:** 2023-05-09

**Authors:** Gaurav K. Deshwal, Laura G. Gómez-Mascaraque, Mark Fenelon, Thom Huppertz

**Affiliations:** 1 Department of Food Chemistry and Technology, Teagasc Food Research Centre, P61 C996 Fermoy, Ireland; gaurav.deshwal@wur.nl (G.K.D.); laura.mascaraque@teagasc.ie (L.G.G.-M.); mark.fenelon@teagasc.ie (M.F.); 2 Department of Agrotechnology and Food Sciences, Wageningen University, Bornse Weilanden 9, 6708 WG Wageningen, The Netherlands; 3 Dairy Technology Division, ICAR-National Dairy Research Institute, Karnal 132001, India; 4 FrieslandCampina, Stationsplein 4, 3818 LE Amersfoort, The Netherlands

**Keywords:** ICP-OES, cheese, digestion, mineral analysis, dry ashing

## Abstract

For sample preparation prior to mineral analysis, microwave digestion (~2 h) is quicker and requires lower acid volume as compared to dry (6–8 h) and wet digestion (4–5 h). However, microwave digestion had not yet been compared systematically with dry and wet digestion for different cheese matrices. In this work, the three digestion methods were compared for measuring major (Ca, K, Mg, Na and P) and trace minerals (Cu, Fe, Mn and Zn) in cheese samples using inductively coupled plasma optical emission spectrometry (ICP-OES). The study involved nine different cheese samples with moisture content varying from 32 to 81% and a standard reference material (skim milk powder). For the standard reference material, the relative standard deviation was lowest for microwave digestion (0.2–3.7%) followed by dry (0.2–6.7%) and wet digestion (0.4–7.6%). Overall, for major minerals in cheese, strong correlation was observed between the microwave and the dry and wet digestion methods (R^2^ = 0.971–0.999), and Bland–Altman plots showed best method agreement (lowest bias), indicating the comparability of all three digestion methods. A lower correlation coefficient, higher limits of agreement and higher bias of minor minerals indicate possibilities of measurement error.

## 1. Introduction

Milk and dairy products make important contributions to intake for many minerals in diets worldwide. For instance, in the Netherlands, dietary intake from dairy products is ~58% for Ca, ~32% for P, ~23% for Zn, ~17% for K and Na and ~15% for Mg [1]. Similar contributions from dairy products to overall dietary intake are also found in other countries, as, e.g., recently reported for Ca [2]. The importance of dairy products to the dietary intake of minerals makes the accurate determination of the content of minerals in dairy products essential. Atomic absorption spectroscopy (AAS) and inductively coupled plasma-optical emission spectrometry (ICP-OES) are considered standard methods for mineral analysis in dairy products. Both techniques require prior sample digestion, which is normally performed by dry ashing or wet ashing [3,4]. Dry ashing consists of the heating, charring and combustion of food samples at 550 °C for no less than 6 h in acid-soaked crucibles [3]. The acid soaking of the crucibles also requires 4 to 6 h, including drying, and is essential to remove residual minerals [5]. Wet ashing commonly involves the boiling of food samples in concentrated nitric (HNO_3_) acid under atmospheric conditions in a flask covered with watch glass until the samples become colorless [6]. As a result, both of these methods of sample digestion require one day for sample preparation. 

The International Organization for Standardization (ISO) and International Dairy Federation (IDF) provide standards for the determination of minerals and trace elements in milk, milk products, infant formula and adult nutritionals, using inductively coupled plasma mass spectrometry (ICP-MS) (ISO21424/IDF243, 2018) [4] and inductively coupled plasma atomic emission spectrometry (ICP-AES) (ISO15151/IDF229, 2018), with microwave digestion as the method for sample digestion. The microwave-assisted wet digestion system involves the heating of the sample and acid mix in polytetrafluoroethylene tubes using a microwave. The digestion procedure details in both the standards are different and state that process parameters require amendment according to the type and size of sample.

Numerous research studies describe the measurement of minerals in cheese using ICP-OES, such as heavy metal residue in dry-ashed cheese [7], the chemometric classification of Brazilian artisanal cheese after microwave digestion [8], trace metals in wet-digested cheese samples packaged in plastic and tin containers [6] and sodium content in retail Cheddar, mozzarella and processed cheese [9]. All the reported studies have used either microwave digestion, dry digestion, or wet digestion only as the sample preparation method prior to the analysis of cheese samples. Some studies report the comparison of microwave digestion and dry ashing prior to determining sodium content in blue cheese [5] and mozzarella cheese [10]. Both the studies reported equivalency between microwave-accelerated digestion and dry ashing for measuring sodium in these cheeses. However, the suitability of these digestion methods to other cheese matrices with varying major and trace mineral elements is uncertain. The present study aimed to compare the suitability of different digestion procedures (microwave digestion, dry digestion and wet digestion) for determining major and trace minerals in a range of different cheese varieties.

## 2. Results and Discussion

### 2.1. Effect of Digestion Methods on Mineral Levels in the Standard Reference Material

Different standard reference materials for minerals have been used in the literature, including tea, rice flour (GBW10010), wheat flour (1567a) and orchard leaves (1571) [11]. However, similar to [12], we selected skim milk powder as the standard reference material since our study was focused on dairy products. All the measured values of different minerals (Ca, K, Mg, P, Cu, Fe and Mn) in the standard reference material (skim milk powder) after the three different digestion methods did not differ significantly (at 5% level of significance), except for sodium and zinc (Table 1). Furthermore, the measured values were in good agreement with the certified values (Table 1). The sodium content obtained using ICP-OES after the wet digestion (4.62 g/kg) method was significantly (*p* < 0.05) higher than after dry (4.18 g/kg) and microwave digestion (4.19 g/kg). Similarly, the zinc content after wet digestion was also significantly (*p* < 0.05) higher than after dry and microwave digestion (Table 1). The sodium and zinc values after microwave and dry digestion were non-significantly different (*p* > 0.05) and very close to the certified value (Table 1).

The precision of the digestion methods for the standard reference material was evaluated by comparing the relative standard deviations of dry, wet and microwave digestion methods as presented in Table 1. The %RSD of the microwave digestion method for all the elements was the lowest, followed by dry and wet digestion. For microwave digestion, the %RSD was well below 5%, which is considered as the acceptable precision range [13]. Correspondingly, for Cu, Fe, Mn and Zn, all the three digestion procedures showed slightly higher %RSD ranging between 0.89 and 3.67%, 2.40 and 6.66% and 2.00 and 7.58%, respectively, indicating slightly lower precision. In addition, microwave digestion showed the lowest %RSD for trace minerals (Cu, Fe, Mn and Zn) compared to dry and wet digestion. Asendorf et al. [12] also reported 11.2%, 2.4% and 2.3% RSD for Fe, Cu and Mo in the standard reference material (skim milk powder BCR-063R) analyzed by ICP-OES after microwave digestion. Chand and Prasad [14] assessed the precision of acid digestion and the alkaline fusion method for heavy metal analysis in marine sediments as percentage relative standard deviation and considered <20% RSD as acceptable, which was well above the values obtained in our work.

### 2.2. Determination of Mineral Levels in Cheeses: Comparison of Digestion Methods

The mineral content of selected cheese samples varied over a broad range. For example, Ca content ranged from 3.18 g/kg for mozzarella cheese to 10.53 g/kg for Emmental cheese. Cheese samples with lower moisture content, especially those below 41% moisture, contained more Ca than cheeses with higher moisture content, except the processed cheese samples. As reported by [15], a higher amount of Ca was measured in hard cheese varieties, which is consistent with the present results. Processed cheese triangles (PT-72) contained almost twice (11.10 g/kg) the amount of phosphorus in comparison to natural cheese samples (Cheddar cheese, 15 g/kg). This was on account of the phosphate salts (polyphosphates and calcium phosphate) added in processed cheese formulations based on the nutritional label of the product. The general trend of major minerals in natural and processed commercial cheese samples was Ca > Na/P > K > Mg. As previously reported, the major mineral content in cheese showed a similar trend (Ca > Na/P > K > Mg) but the influences of moisture and species (cow, sheep and buffalo milk) have also been signified [16].

Our study showed non-significant differences between the digestion techniques (microwave, wet and dry digestion) for the evaluation of most of the major minerals using ICP-OES as per t-test at 5% level of significance (Table 2). However, some exceptions existed, with significant differences (*p* < 0.05) between the wet and the other two digestion techniques (microwave and dry) for major minerals (Ca content in CC-39, K content in EM-41, Mg content in MO-32, Na content in FC-57 and P content in PB-51) (Table 2). The reason for greater differences between the wet digestion and the other two digestion methods could be the loss of analytes by evaporation or incomplete sample dissolution [11].

Regarding trace minerals, several samples showed non-significant differences (*p* > 0.05) using t-test in their values (Table 2) but no concrete conclusion about the preferred digestion techniques prior to the ICP-OES-based analysis of trace minerals in cheese could be drawn. It could possibly be due to the higher dilution factor applied for cheese sample preparation owing to which the detection limits for trace minerals are not correctly achieved using ICP-OES. In the present study, the limits of detection for Cu, Fe, Mn and Zn were 0.047 mg/kg, 0.031 mg/kg, 0.018 mg/kg and 0.012 mg/kg, respectively. Asendorf et al. [12] showed that the amounts of selenium, lead and arsenic in infant formulae and milk powders were below the method detection limit using ICP-OES.

**Figure 1:**
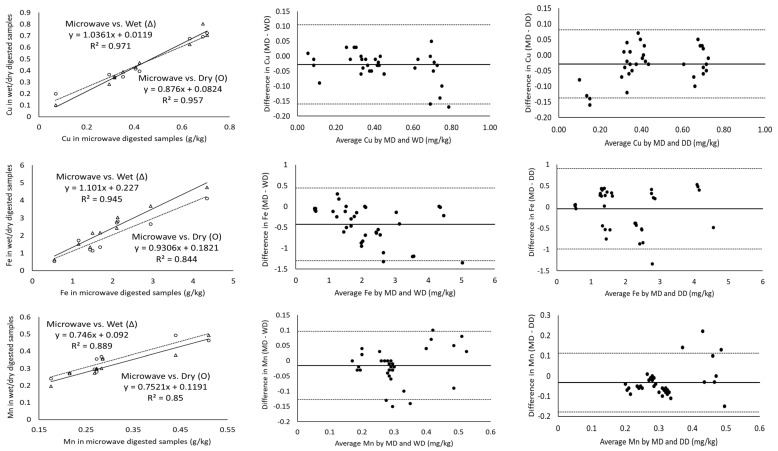

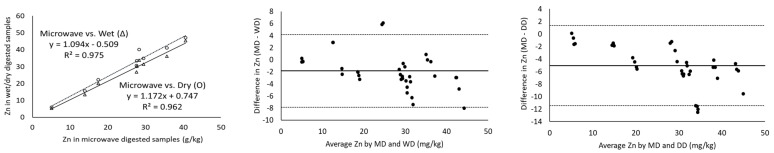
Correlation of minor minerals as measured by ICP-OES for microwave-wet (Δ) and microwave-dry (O) digested cheese samples (column 1), Bland–Altman plots of the minor mineral measurements by microwave digestion (MD) and wet digestion (WD) (column 2) and Bland–Altman plots of minor mineral measurements by microwave digestion (MD) and dry digestion (DD) (column 3). For all the Bland–Altman plots, the solid black line represents the observed mean agreement (bias) between methods and dashed lines above and below the solid black line represent the upper and lower limits of agreement, respectively. (Limits of agreement = bias ± (1.96 × standard deviation)).

**Figure 2:**
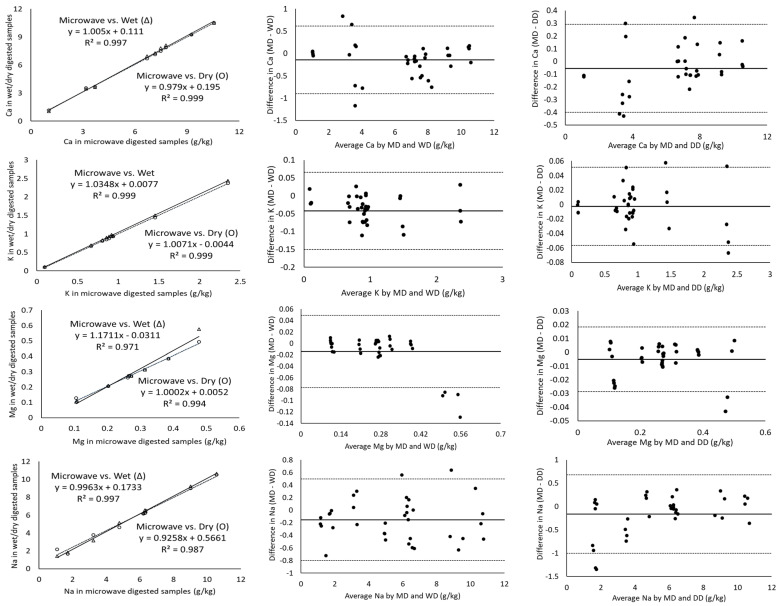

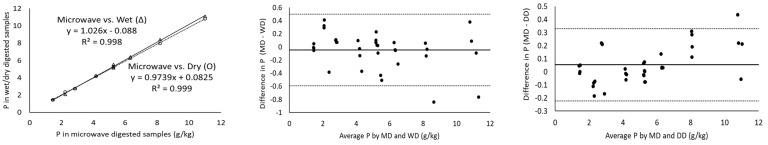
Correlation of major minerals as measured by ICP-OES for microwave vs. wet (Δ) and microwave vs. dry (O) digested cheese samples (column 1), Bland–Altman plots of the major mineral measurements by microwave digestion (MD) and wet digestion (WD) (column 2) and Bland–Altman plots of the major mineral measurements by microwave digestion (MD) and dry digestion (DD) (column 3). For all the Bland–Altman plots, the solid black line represents the observed mean agreement (bias) between methods and dashed lines above and below the solid black line represent the upper and lower limits of agreement, respectively. (Limits of agreement = bias ± (1.96 × standard deviation)).

The magnitude of the correlation coefficient is a measure of the changeability of one variable explained by a shift in the other variable and an R^2^ value of 1 indicates a perfect fit [17]. Considering the measurement of major minerals present in cheeses including Ca, K, Mg, Na and P using ICP-OES, the digestion of the samples with microwave vs. wet and microwave vs. dry digestion showed a correlation coefficient greater than 0.971 in all cases (Table 3). In addition, Ca, K and P showed a strong correlation coefficient of ~0.99 for all the three digestion methods (Figure 1). Wet digestion (R^2^-0.971) showed a slightly lower correlation coefficient than dry digestion (R^2^-0.994) for the measurement of Mg (Table 3) in comparison to microwave digestion. On the other hand, minor minerals (Cu, Fe, Mn and Zn) showed lower correlation coefficients of 0.844–0.975 for microwave vs. both dry and wet digestion methods, individually (Table 3). For example, the Mn and Fe measurements of microwave-digested samples showed R^2^ measurements of 0.850 and 0.844 with dry digestion, respectively. While the relationship between the measurement of Fe and Mn was weak, the Bland–Altman plots showed random errors (Figure 2). Since the correlation coefficient measures the level of association between two variables, a high correlation coefficient does not necessarily refer to good agreement between two methods [17]. Therefore, the correlation of the data was not sufficient to conclude the comparability of dry and wet digestion methods with microwave digestion for both major and minor minerals. Further, the comparison and evaluation of agreement between different digestion methods was performed by Bland–Altman plots.

All the mineral measurements showed a negative bias for microwave vs. wet digestion and microwave vs. dry digestion, except phosphorus for microwave vs. dry digestion, which means that, on average, wet or dry digestion shows higher minerals content than microwave digestion (Table 3). For P, microwave and dry digestion showed a bias of 0.053. The Bland–Altman plot does not indicate the suitability of using a method but simply quantifies the upper and lower limits of agreement within which 95% of the differences between one measurement and another should lie [18]. The Bland–Altman plot analysis for Ca showed a mean difference (microwave minus wet digestion) of −0.141 g/kg and the ranges for limits of agreements (from bias-1.96 × SD to bias-1.96 × SD) were −0.896 to 0.613 g/kg, which were sufficiently narrow. These limits of agreement indicate that measured values of Ca by wet digestion may be 0.613 g/kg above or 0.896 g/kg below microwave-digested samples. Cheese generally contains a higher amount of Ca in comparison to milk and yoghurt [19]; therefore, ± (0.613–0.896) g/kg would not be significant, but for dairy products with Ca in trace amounts a difference of ± (0.613–0.896) g/kg would be important. The mean differences in P measurement for wet-digested cheese samples compared to microwave-digested were +0.497 and −0.592 g/kg, respectively (Table 3). Similarly, other major minerals (K, Mg and Na) had narrow ranges of agreement limits, suggesting the good agreement of microwave digestion with dry and wet digestion methods individually. 

For the minor minerals, the limits of agreement were slightly higher and no specific trend was observed for any of the digestion methods. Of all the minor minerals, Zn showed the highest bias (−1.85 and −5.05 mg/kg) and LOA (−11.47 and 1.35 mg/kg) (Table 3; Figure 1). The measured values for minor minerals in cheese fell within the LOA (Figure 1 and Figure 2) but the samples showed a lower correlation coefficient (Table 3) and significant differences in the values (*p* < 0.05) (Table 2). Cu (R^2^-0.971), Fe (R^2^-0.945) and Zn (R^2^-0.975) showed higher correlation coefficients for microwave vs. wet digestion, but wider LOAs indicate that large measurement errors could occur. These differences could be attributed to the ineffectiveness of ICP-OES for measuring minor minerals in cheese. An inter-lab collaborative study focused on the determination of minerals and trace elements in dairy products reported acceptable accuracy and precision for Ca, K, Mg, Na and P, but not for Cu, Fe, Zn and Mn due to their lower concentrations in dairy products [19]. Inductively coupled plasma-mass spectroscopy (ICP-MS) equipped with a collision/reaction cell showed better accuracy and reproducibility in testing the lower concentrations of Cu, Fe, Zn and Mn in dairy products (cheese, butter, infant formula, dairy powders) [20]. Both the above studies suggested the use of ICP-MS or graphite furnace atomic absorption spectroscopy (GF-AAS) for the quantification of these trace minerals.

The solid line in Bland–Altman plots represents the observed mean agreement (bias) between two methods (microwave vs. wet digestion in Figure 2 and microwave vs. dry digestion in Figure 1) for each sample, while the dashed lines above and below the mean line represent the upper and lower limits of agreement. The Bland–Altman plots showed no systematic error in the relationship between the ICP-OES-based measurement of mineral contents in the cheese samples digested by microwave, wet and dry digestion (Figure 1 and Figure 2). The Bland–Altman plots for Ca showed all the measured values above 6 g/kg to be clustered near the mean difference line, while the values below 4 g/kg Ca were more scattered and three values were outside the upper and lower LOA for both microwave vs. wet digestion (Figure 2) and microwave vs. dry digestion (Figure 1). This signifies the equivalent suitability of microwave, dry and wet digestion for digesting cheese samples with Ca concentrations above 6 g/kg. The mean differences in the K measurement between microwave and wet digestion were within ±1.96 × standard deviations (Figure 2). Contrarily, Mg measurements showed measured values outside the LOA at concentrations above 0.5 g/kg, suggesting the suitability of the three digestion methods for measuring lower concentrations of Mg in cheese. In comparison to wet-digested samples, microwave-digested cheese samples had slightly overestimated Mg for concentrations below 0.4 g/kg and underestimated Mg at concentrations above 0.4 g/kg (Figure 2).

## 3. Materials and Methods

### 3.1. Sampling

Nine different cheese samples, available in supermarkets in Fermoy (Ireland), were collected; the samples included cheeses with moisture content ranging from approximately 31 to 81%: Manchego cheese (MO-32), mature white Cheddar (CC-39), French Emmental (EM-41), Halloumi cheese (HC-48), processed cheese block (PB-51), Greek feta (FC-57), Irish buffalo mozzarella (MC-65), processed cheese triangles (PT-72) and fat-free cottage cheese (CO-81). Skimmed milk powder (ERM-BD151, Sample No: 1169, European Commission, Joint Research Centre, Directorate F-Health, Consumers and Reference Materials, Geel, Belgium) having a certified mineral content was used as standard reference material in this study. 

### 3.2. Sample Digestion Procedures

Three different digestion procedures were used to digest the organic material and isolate the inorganic mineral fraction as described below. For testing the accuracy of digestion procedures, standard reference material (skimmed milk powder) was treated in the same way as described for cheese samples. All the glassware used in the digestion procedures was rinsed with 5% HNO_3_ and ultra-pure water at least twice. TraceSELECT grade (>69% purity) of HNO_3_ was used for all the digestion methods.

#### 3.2.1. Dry Digestion

Approximately 1 g of cheese sample was weighed accurately and ashed in gravimetric oven (TGA701, LECO Corporation, St. Joseph, MI, USA) at 550 ± 5 °C until constant weight was reached [3]. The ashed samples were subsequently mixed with 5 mL of concentrated HNO_3_, filtered through glass wool and the volume of the filtrate was made up to 100 mL using deionized water. 

#### 3.2.2. Wet Digestion

Cheese samples (approx. 1 g) were accurately weighed and mixed with 15 mL HNO_3_ and digested at 130–140 °C for 4–5 h using a hot-plate in a fume hood. The point at which reddish brown color fumes ceased and the sample solution became colorless was considered as the end point of the digestion process [6]. The samples were filtered through glass wool and the filtrate volume was made up to 100 mL using deionized water.

#### 3.2.3. Microwave Digestion

About 1 g of cheese sample was accurately weighed in Teflon cylindrical tubes and a volume of 5 mL concentrated HNO_3_ (CAS-No. 7697-37-2, Fisher Scientific Ltd., Loughborough, UK) of TraceSELECT grade (>69% purity) was added. The microwave digestion program equivalent to [4] was followed. Samples were digested in two stages at 180 °C (1600 W) and 200 °C (1600 W) for 20 min each using a microwave digester (MARS6, One touch technology, CEM Corporation, Matthews, NC, USA). Finally, the digested samples were allowed to cool down for 20 min and transferred to volumetric flask for making up the volume to 100 mL using deionized water.

### 3.3. ICP-OES Analysis

The ICP-OES analysis was performed using an Agilent 5110 synchronous vertical dual view ICP-OES analyzer (Agilent Technologies, Santa Clara, CA, USA). The instrument was calibrated with 9 different element standards (ICP standards prepared in 2–5% HNO_3_ matrix, REICCAL 10CR5, Reagecon, Shannon, Ireland) by setting correlation coefficient limit at ≥0.999. Yttrium (Y) and cesium (Cs) solution (0.4 mL Y and 10 mL Cs made up to 100 mL using 5% HNO_3_) were used as an internal standard and ionization buffer, respectively, to minimize easily ionizable element effect and to correct any signal drift due to physical and chemical interference. Some food materials may contain a significant amount of easily ionizable element (e.g., Ca, K and Na), which provides a substantial source of electrons in plasma. The effect of variable concentration of an easily ionizable element in all the samples and standards has been minimized by ionization buffer [19]. The instrument running conditions and wavelength of the spectrometer for each element were similar to those mentioned by [6]. Calibration was performed after every 20 samples in every single run using five different levels of standard solutions.

### 3.4. Statistical Analysis

For standard reference material, the relative standard deviation (calculated as the ratio of standard deviation to mean) was evaluated as a measure of the deviation of the obtained results around the mean. The statistical differences of the experimental data at 5% significance level were assessed using SPSS (IBM SPSS, 2020; version 27) software following Duncan’s test. Correlation analysis identified the association between the individual digestion methods based on their correlation coefficient (R^2^), which measures the closeness of the observations to the regression line [17]. The linear relationship between each two methods was thus obtained by plotting values obtained for each component by one method against their corresponding values from the second method and obtaining the regression line. Identification of outliers was based on the visual inspection of data using scatter diagrams. However, correlation coefficients of one method against the other do not inform the between-method differences [17].

Bland–Altman difference plots were used to evaluate agreement between two quantitative measurements by studying the mean difference and constructing limits of agreement. A purported acceptable limit of agreement was considered as 95% of the measured samples lying within ± 1.96 × standard deviation from the mean differences. Bias was calculated as the mean of the difference between two individual measurement methods (microwave digestion or dry digestion or wet digestion). The upper and lower limits of agreement (LOA) were calculated $\;\left(\mathrm{LOA}=\mathrm{Bias}\;\pm \left(1.96\times \mathrm{SD}\right)\right)$, and are illustrated using Bland–Altman plots [18].

## 4. Conclusions

This is the first study that has systematically compared three different digestion methods for cheese prior to mineral analysis using ICP-OES. Microwave, dry and wet digestion were sufficiently accurate in digesting the cheese sample for mineral analysis. However, the choice of digestion method should take into account the requirements for precision and accuracy. While the digestion method correlation was high for major minerals, the mean difference values were within the limits of agreement for major and minor minerals. The limits of agreement for minor minerals were high, which shows possibilities of significant differences in their measured values using differently digested cheese samples. The microwave digestion time-temperature profile recommended by [4] was adequate to completely digest the cheese matrix with varying moisture content (32–81%). This research also provides important information on the concentration of macro- and micro-elements in a variety of commercial cheese samples. In future, studies on the validation of trace mineral analysis in cheese samples using ICP-MS with different sample digestion techniques should be undertaken.

## Figures and Tables

**Table 1:** Accuracy assessment through the mineral analysis (in mg/100 g) of the skim milk powder certified reference material (ERM-BD151). Values for Ca, K, Mg, Na and P are in g/kg and for Cu, Fe, Mn and Zn are in mg/kg.

Element	Certified Value	Dry Digestion	%RSD	Recovery (%)	Wet Digestion	%RSD	Recovery (%)	Microwave Digestion	%RSD	Recovery (%)
Ca	13.9 ± 0.70	13.88 ± 0.23 ^a^	0.16	99.85	13.82 ± 0.53 ^a^	0.38	99.37	13.87 ± 0.24 ^a^	0.17	99.79
K	17.0 ± 0.80	15.99 ± 0.36 ^a^	2.27	94.06	16.52 ± 0.98 ^a^	5.90	97.15	16.16 ± 0.12 ^a^	0.77	95.07
Mg	1.26 ± 0.07	1.22 ± 0.04 ^a^	2.02	97.11	1.25 ± 0.02 ^a^	1.96	99.36	1.24 ± 0.04 ^a^	0.34	98.21
Na	4.19 ± 0.23	4.18 ± 0.12 ^a^	2.93	99.66	4.04 ± 0.27 ^b^	5.89	96.52	4.19 ± 0.14 ^a^	0.34	99.88
P	11.0 ± 0.60	10.02 ± 0.31 ^a^	3.04	91.11	9.86 ± 0.29 ^a^	2.95	89.60	9.98 ± 0.10 ^a^	1.03	90.76
Cu	5.00 ± 0.23	4.50 ± 0.10 ^a^	2.40	89.60	4.60 ± 0.10 ^a^	2.00	92.85	4.70 ± 0.10 ^a^	1.00	93.00
Fe	53.0 ± 4.00	55.4 ± 3.70 ^a^	6.66	104.59	53.1 ± 1.20 ^a^	2.22	100.19	51.5 ± 0.50 ^a^	0.89	97.22
Mn	0.29 ± 0.03	0.34 ± 0.02 ^a^	4.79	118.10	0.35 ± 0.03 ^a^	7.58	122.41	0.36 ± 0.02 ^a^	3.40	124.13
Zn	44.9 ± 2.30	46.7 ± 1.90 ^ab^	4.16	114.98	51.6 ± 3.90 ^b^	7.57	104.01	44.7 ± 1.60 ^a^	3.67	99.48

^ab^ Values for different digestion methods with different superscripts within a row are significantly different (*p* < 0.05). Average ± S.D (*n* = 4).

**Table 2:** Mineral concentrations in Manchego cheese (MO-32), mature white Cheddar (CC-39), French Emmental (EM-41), Halloumi cheese (HC-48), processed cheese block (PB-51), Greek feta (FC-57), Irish buffalo mozzarella (MC-65), processed cheese triangle (PT-72) and fat-free cottage cheese (CO-81) (major minerals in g/kg and trace minerals in mg/kg) determined by inductively coupled plasma-optical emission spectroscopy (ICP-OES) following dry, wet and microwave digestion of samples.

Sample	Digestion	Major Minerals in Cheese (g/kg)					Trace Minerals in Cheese (mg/kg)			
		Ca	K	Mg	Na	P	Cu	Fe	Mn	Zn
MO-32	Wet	8.12 ± 0.37 ^a^	0.95 ± 0.021 ^a^	0.58 ± 0.033 ^b^	6.23 ± 0.43 ^a^	5.51 ± 0.23 ^a^	0.80 ± 0.049 ^b^	1.52 ± 0.26 ^b^	0.38 ± 0.004 ^a^	30.15 ± 0.54 ^b^
	Microwave	7.78 ± 0.05 ^a^	0.94 ± 0.012 ^a^	0.48 ± 0.022 ^a^	6.21 ± 0.09 ^a^	5.26 ± 0.01 ^a^	0.69 ± 0.013 ^a^	1.15 ± 0.08 ^a^	0.44 ± 0.021 ^b^	27.64 ± 0.18 ^a^
	Dry	7.88 ± 0.05 ^a^	0.93 ± 0.012 ^a^	0.49 ± 0.001 ^a^	6.16 ± 0.10 ^a^	5.28 ± 0.06 ^a^	0.69 ± 0.033 ^a^	1.71 ± 0.11 ^b^	0.49 ± 0.046 ^b^	33.41 ± 0.95 ^c^
CC-39	Wet	7.80 ± 0.09 ^b^	0.93 ± 0.022 ^b^	0.31 ± 0.007 ^a^	6.41 ± 0.34 ^a^	5.15 ± 0.07 ^a^	0.34 ± 0.028 ^a^	4.74 ± 0.56 ^a^	0.36 ± 0.047 ^b^	36.18 ± 1.46 ^a^
	Microwave	7.47 ± 0.22 ^a^	0.88 ± 0.011 ^a^	0.31 ± 0.002 ^a^	6.34 ± 0.08 ^a^	5.26 ± 0.04 ^b^	0.32 ± 0.011 ^a^	4.34 ± 0.01 ^a^	0.29 ± 0.005 ^a^	35.64 ± 0.26 ^a^
	Dry	7.49 ±0. 09 ^a^	0.88 ± 0.009 ^a^	0.31 ± 0.003 ^a^	6.49 ± 0.06 ^a^	5.25 ± 0.02 ^b^	0.34 ± 0.024 ^a^	4.11 ± 0.40 ^a^	0.36 ± 0.014 ^b^	41.11 ± 0.88 ^b^
EM-41	Wet	10.48 ± 0.01 ^a^	0.90 ± 0.027 ^b^	0.39 ± 0.004 ^a^	1.82 ± 0.12 ^a^	6.40 ± 0.15 ^a^	0.47 ± 0.022 ^b^	3.04 ± 0.22 ^b^	0.30 ± 0.007 ^a^	45.31 ± 1.80 ^b^
	Microwave	10.53 ± 0.04 ^a^	0.85 ± 0.023 ^a^	0.39 ± 0.001 ^a^	1.72 ± 0.06 ^a^	6.32 ± 0.03 ^a^	0.42 ± 0.004 ^ab^	2.12 ± 0.10 ^a^	0.28 ± 0.004 ^a^	40.62 ± 0.28 ^a^
	Dry	10.52 ± 0.05 ^a^	0.85 ± 0.028 ^a^	0.38 ± 0.002 ^a^	1.66 ± 0.06 ^a^	6.27 ± 0.06 ^a^	0.39 ± 0.037 ^a^	2.82 ± 0.08 ^b^	0.37 ± 0.014 ^b^	47.07 ± 1.59 ^b^
HC-48	Wet	6.95 ± 0.22 ^a^	0.83 ± 0.061 ^a^	0.27 ± 0.011 ^a^	10.65 ± 0.32 ^a^	4.27 ± 0.16 ^a^	0.28 ± 0.032 ^a^	2.42 ± 0.32 ^ab^	0.20 ± 0.015 ^a^	31.46 ± 1.36 ^b^
	Microwave	6.70 ± 0.04 ^a^	0.80 ± 0.011 ^a^	0.27 ± 0.001 ^a^	10.55 ± 0.08 ^a^	4.16 ± 0.02 ^a^	0.30 ± 0.026 ^a^	2.10 ± 0.01 ^a^	0.18 ± 0.005 ^a^	29.41 ± 0.17 ^a^
	Dry	6.70 ± 0.05 ^a^	0.81 ± 0.032 ^a^	0.27 ± 0.002 ^a^	10.53 ± 0.21 ^a^	4.18 ± 0.04 ^a^	0.37 ± 0.018 ^b^	2.73 ± 0.41 ^b^	0.24 ± 0.014 ^b^	34.92 ± 0.70 ^c^
PB-51	Wet	7.28 ± 0.05 ^b^	0.97 ± 0.018 ^b^	0.27 ± 0.011 ^a^	9.23 ± 0.50 ^a^	8.42 ± 0.37 ^b^	0.43 ± 0.011 ^b^	2.17 ± 0.27 ^c^	0.30 ± 0.015 ^b^	33.74 ± 1.49 ^b^
	Microwave	7.15 ± 0.04 ^a^	0.92 ± 0.012 ^a^	0.26 ± 0.002 ^a^	9.02 ± 0.28 ^a^	8.18 ± 0.04 ^ab^	0.41 ± 0.005 ^a^	1.69 ± 0.07 ^b^	0.27 ± 0.004 ^a^	28.30 ± 0.15 ^a^
	Dry	7.14 ± 0.08 ^a^	0.93 ± 0.020 ^a^	0.26 ± 0.006 ^a^	9.00 ± 0.20 ^a^	7.96 ± 0.04 ^a^	0.42 ± 0.008 ^ab^	1.34 ± 0.13 ^a^	0.28 ± 0.012 ^ab^	40.19 ± 0.40 ^c^
FC-57	Wet	3.64 ± 0.49 ^a^	0.70 ± 0.019 ^a^	0.21 ± 0.006 ^a^	5.13 ± 0.06 ^b^	2.76 ± 0.04 ^a^	0.71 ± 0.026 ^a^	1.35 ± 0.19 ^a^	0.27 ± 0.023 ^a^	13.42 ± 2.32 ^a^
	Microwave	3.69 ± 0.03 ^a^	0.66 ± 0.012 ^a^	0.20 ± 0.002 ^a^	4.77 ± 0.05 ^a^	2.85 ± 0.02 ^a^	0.71 ± 0.011 ^a^	1.44 ± 0.05 ^a^	0.27 ± 0.008 ^a^	13.86 ± 0.17 ^a^
	Dry	3.68 ± 0.23 ^a^	0.67 ± 0.018 ^a^	0.22 ± 0.002 ^a^	4.64 ± 0.18 ^a^	2.73 ± 0.04 ^a^	0.72 ± 0.019 ^a^	1.20 ± 0.10 ^a^	0.30 ± 0.02 ^a^	15.59 ± 0.20 ^a^
MC-65	Wet	3.45 ± 0.69 ^a^	0.11 ± 0.017 ^a^	0.11 ± 0.009 ^a^	3.77 ± 0.08 ^b^	2.07 ± 0.28 ^a^	0.39 ± 0.019 ^b^	2.15 ± 0.29 ^c^	0.27 ± 0.086 ^a^	26.79 ± 5.34 ^a^
	Microwave	3.18 ± 0.10 ^a^	0.09 ± 0.003 ^a^	0.11 ± 0.003 ^a^	3.24 ± 0.12 ^a^	2.23 ± 0.04 ^a^	0.35 ± 0.004 ^ab^	1.50 ± 0.04 ^b^	0.22 ± 0.005 ^a^	27.61 ± 0.20 ^a^
	Dry	3.54 ± 0.09 ^a^	0.10 ± 0.006 ^a^	0.10 ± 0.005 ^a^	3.15 ± 0.17 ^a^	2.35 ± 0.04 ^a^	0.35 ± 0.028 ^a^	1.14 ± 0.08 ^a^	0.27 ± 0.007 ^a^	30.62 ± 2.28 ^a^
PT-72	Wet	9.30 ± 0.15 ^a^	2.44 ± 0.110 ^a^	0.27 ± 0.005 ^a^	6.58 ± 0.10 ^b^	11.10 ± 0.41 ^a^	0.63 ± 0.106 ^a^	3.68 ± 0.47 ^b^	0.30 ± 0.016 ^b^	19.96 ± 0.45 ^b^
	Microwave	9.23 ± 0.03 ^a^	2.35 ± 0.016 ^a^	0.27 ± 0.001 ^b^	6.30 ± 0.19 ^a^	11.00 ± 0.08 ^a^	0.63 ± 0.046 ^a^	2.94 ± 0.02 ^a^	0.27 ± 0.013 ^a^	17.47 ± 0.06 ^a^
	Dry	9.23 ± 0.10 ^a^	2.37 ± 0.032 ^a^	0.27 ± 0.003 ^b^	6.23 ± 0.05 ^a^	10.80 ± 0.17 ^a^	0.68 ± 0.033 ^a^	2.65 ± 0.08 ^a^	0.36 ± 0.011 ^c^	22.25 ± 0.74 ^c^
CO-81	Wet	1.04 ± 0.04 ^a^	1.50 ± 0.047 ^a^	0.11 ± 0.004 ^a^	1.41 ± 0.25 ^a^	1.46 ± 0.02 ^a^	0.10 ± 0.039 ^a^	0.62 ± 0.002 ^b^	0.49 ± 0.028 ^b^	5.25 ± 0.23 ^a^
	Microwave	1.04 ± 0.01 ^a^	1.45 ± 0.004 ^a^	0.11 ± 0.001 ^b^	1.08 ± 0.03 ^a^	1.45 ± 0.03 ^a^	0.07 ± 0.007 ^a^	0.55 ± 0.001 ^a^	0.51 ± 0.043 ^b^	5.06 ± 0.14 ^a^
	Dry	1.15 ± 0.06 ^b^	1.44 ± 0.034 ^a^	0.13 ± 0.002 ^a^	2.19 ± 0.20 ^b^	1.43 ± 0.03 ^a^	0.20 ± 0.034 ^b^	0.53 ± 0.003 ^a^	0.36 ± 0.053 ^a^	6.01 ± 0.64 ^b^

^abc^ Values with different superscripts within a column for a particular cheese sample are significantly different (*p* < 0.05). (Average ± SD; *n* = 4).

**Table 3:** Correlation coefficient (R^2^), bias and limits of agreement (LOA) for the determination of minerals by inductively coupled plasma-optical emission spectroscopy (ICP-OES) following dry, wet and microwave digestion of cheese samples.

Minerals	Method	R^2^	Bias	SD ^#^	Lower LOA *	Upper LOA *
Ca	Microwave-Wet	0.997	−0.141	0.385	−0.896	0.613
	Microwave-Dry	0.999	−0.055	0.176	−0.400	0.290
K	Microwave-Wet	0.999	−0.042	0.055	−0.151	0.066
	Microwave-Dry	0.999	−0.003	0.028	−0.057	0.051
Mg	Microwave-Wet	0.971	−0.014	0.032	−0.078	0.049
	Microwave-Dry	0.994	−0.001	0.012	−0.029	0.018
Na	Microwave-Wet	0.997	−0.153	0.331	−0.802	0.496
	Microwave-Dry	0.987	−0.160	0.429	−1.002	0.681
P	Microwave-Wet	0.998	−0.047	0.278	−0.592	0.497
	Microwave-Dry	0.999	0.053	0.141	−0.223	0.329
Cu	Microwave-Wet	0.971	−0.030	0.07	−0.159	0.105
	Microwave-Dry	0.957	−0.03	0.06	−0.138	0.081
Fe	Microwave-Wet	0.945	−0.428	0.445	−1.301	0.446
	Microwave-Dry	0.844	−0.045	0.483	−0.991	0.902
Mn	Microwave-Wet	0.889	−0.015	0.057	−0.127	0.097
	Microwave-Dry	0.850	−0.033	0.074	−0.179	0.113
Zn	Microwave-Wet	0.975	−1.85	3.07	−7.85	4.16
	Microwave-Dry	0.962	−5.05	3.27	−11.47	1.35

* LOA—Limit of agreement also defined as 95% confidence interval. # SD—Standard Deviation.

## Data Availability

Not applicable.
